# Perinatal Health Statistics as the Basis for Perinatal Quality Assessment in Croatia

**DOI:** 10.1155/2015/537318

**Published:** 2015-11-29

**Authors:** Urelija Rodin, Boris Filipović-Grčić, Josip Đelmiš, Tatjana Glivetić, Josip Juras, Željka Mustapić, Ruža Grizelj

**Affiliations:** ^1^School of Medicine, University of Zagreb, School of Public Health “A. Štampar”, Croatian Institute of Public Health, Rockefellerova 4, 10000 Zagreb, Croatia; ^2^School of Medicine, University of Zagreb, Clinical Hospital Center Zagreb, Department of Pediatrics, Kišpatićeva 12, 10000 Zagreb, Croatia; ^3^School of Medicine, University of Zagreb, Clinical Hospital Center Zagreb, Department of Obstetrics and Gynecology, Petrova 13, 10000 Zagreb, Croatia; ^4^General Hospital Zabok, Trg Dragutina Domjanića 6, 49210 Zabok, Croatia

## Abstract

*Context*. Perinatal mortality indicators are considered the most important measures of perinatal outcome. The indicators reliability depends on births and deaths reporting and recording. Many publications focus on perinatal deaths underreporting and misclassification, disabling proper international comparisons.* Objective*. Description of perinatal health care quality assessment key indicators in Croatia.* Methods*. Retrospective review of reports from all maternities from 2001 to 2014.* Results*. According to reporting criteria for birth weight ≥500 g, perinatal mortality (PNM) was reduced by 31%, fetal mortality (FM) by 32%, and early neonatal mortality (ENM) by 29%. According to reporting criteria for ≥1000 g, PNM was reduced by 43%, FM by 36%, and ENM by 54%. PNM in ≥22 weeks' (wks) gestational age (GA) was reduced by 28%, FM by 30%, and ENM by 26%. The proportion of FM at 32–36 wks GA and at term was the highest between all GA subgroups, as opposed to ENM with the highest proportion in 22–27 wks GA. Through the period, the maternal mortality ratio varied from 2.4 to 14.3/100,000 live births. The process indicators have been increased in number by more than half since 2001, the caesarean deliveries from 11.9% in 2001 to 19.6% in 2014.* Conclusions*. The comprehensive perinatal health monitoring represents the basis for the perinatal quality assessment.

## 1. Introduction

Perinatal health care, as well as the other areas of health care, requires the usage of useful indicators for quality assessment and evaluation, which will enable sustainable planning in accordance with limited resources. A weighted sum of all essential indicators, including fetal and maternal, short-term, and long-term outcomes, as well as maternal satisfaction and the impact on future pregnancies and deliveries, would represent the ideal measure of quality [1]. However, all recommended perinatal health indicators cannot be produced and gathered through routine national health statistics system. Some of the indicators are already available in the international databases but not presented by subgroups, which would make them more specific and sensitive perinatal health measurements. According to the World Health Organization (WHO) recommendations for international comparisons, the countries should calculate the perinatal indicators for total births, fetal and early neonatal deaths ≥1000 g birth weight (BW), or ≥28 weeks' (wks) gestational age (GA). Moreover, the inclusion of fetuses and infants weighing 500–999 g or 22–27 completed wks GA in national statistics is recommended by WHO because it improves the coverage of reporting according to criteria for international comparisons and enables better evaluation outcomes [2]. Also, on the European community's research agenda, there was a need for defining measures of maternal and child health care and outcomes for use in evaluating health care and public health programmes. As a part of the EU's Health Monitoring Programme, PERISTAT (Perinatal Statistics) project has been launched in 1999 [3]. The objective of the PERISTAT project is to establish a high quality, innovative, internationally recognized, and sustainable European perinatal health information system. This system's goal is to produce data and analysis on a regular basis for use by national, European, and international stakeholders who make decisions about the health and health care of pregnant women and newborns. PERISTAT scientific advisory committee defined the core perinatal indicators list in order to monitor the perinatal health more precisely. These indicators are sufficient for international comparisons, measuring fetal and infant health outcomes and key interventions implemented to prevent death and morbidity [4, 5].

The aim of this study was to analyze the key indicators for perinatal health care quality assessment in Croatia for the period 2001–2014.

The feasible perinatal indicators for Croatia's perinatal health care assessment were as follows:

- Perinatal outcome indicators: perinatal mortality (PNM), fetal mortality (FM), early neonatal mortality (ENM) by BW subgroups (≥500 g and ≥1000 g) and GA subgroups (≥22 wks and ≥28 wks), and maternal mortality.
- Process indicators: antenatal visits, ultrasound (US) examinations, and caesarean deliveries (CS).

## 2. Material and Methods

### 2.1. Croatian Population Characteristics

According to the 2011 Census, the Croatian Bureau of Statistics (CBS) data, the Croatian population amounted to approximately 4.3 million, with approximately 40,000 deliveries and 50,000 deaths per year with negative natural trend [6]. Sociodemographic characteristics of population by WHO's estimates indicated low birth rate (9.3/1,000), low total fertility rate (1.5/per woman, 15–49 years old), moderate death rate (12.0/1,000), and moderate life expectancy at birth for both sexes (76 years) [7]. Numerous sociodemographic characteristics of mothers remain unknown since CBS collects a limited data set of these data like permanent residence, marital status, parity, and professional birth attendance [6]. According to CBS data in the period 2000–2013, more than 80% of births were from marriages and more than 99% in health institutions [8]. Almost half of all deliveries were first deliveries, 35% second, and 15% third or higher birth order. Concerning the mother age, the deliveries were most common at the age 25–29 (91.8 deliveries per 1,000 females of the same age), followed by the age 30–34 (89.7 deliveries per 1,000 females of this age), and at the age 20–24 (47.5/1,000). The overall average birth age was 30, while the average age at first birth among women was 27 years [6, 9]. About 3% were births from multiple pregnancies [9]. CBS vital statistics data about births are limited to sex, live birth (LB) or stillbirth, and residence. It was a basic reason for routine health statistics system improvement and introduction of new medical birth notification for birth monitoring with a broader set of data in 2001.

Despite efforts for better data entry in health institutions, the Croatian Institute of Public Health (CIPH) as the main producer of routine health statistics disposes of some basic data of newborns like sex, GA, and BW. According to CIPH data, 20,283 males and 19,505 females were born in the year 2014 and there was 1,04 : 1 male : female newborn ratio. In this period, there were 5.24% newborns with BW less than 2500 g: 0.51% newborns with extremely low birth weight (less than 1000 g (ELBW) newborns), 0.47% newborns of BW 1000–1499 g, 1.08% newborns of BW 1500–1999 g, and 3.18% newborns of BW 2000–2499 g [9].

During the war and the postwar period in Croatia, the share of preterm births amounted to 7-8% [10]; in the period 2001–2010, this share decreased to 5.8% [11], while in the period 2010–2014, the increase was present up to 6.2% [12].

### 2.2. Study Design

The reports were retrieved from CIPH for the period 2001–2014, after collecting and processing birth, fetal deaths, and early neonatal deaths data from maternities and neonatal intensive care units (NICUs). These reports derived from hospital data were obtained as a part of the CIPH and Croatian Society of Perinatal Medicine (CSPM) Programme for perinatal health surveillance and reporting. According to these reports, there were 587,356 total births ≥22 wks and 4,633 perinatal deaths ≥22 wks (Table 1).

Perinatal data collecting according to the WHO recommendations by the BW and GA groups has been introduced in Croatian maternities since 2001 by the CIPH, in cooperation with CSPM, through two ways: reports with aggregated birth and perinatal death data by the BW and GA subgroups and individual birth and perinatal death notifications based on hospital discharges [10].

WHO encourages countries to build perinatal monitoring capacity and collect data for key perinatal mortality indicators, relying on the same definitions in order to allow for the comparison of these statistics. The events related to birth, death, and the perinatal period, as well as the reporting requirements for the data from which internationally comparable statistics are drawn, are defined in detail in the International Classification of Diseases, 10th Edition (ICD-10), Instruction Manual, [2].

WHO recommended definition for national purposes [2]:

(1) PNM=Fetal  deaths  and  early  neonatal  deaths≥500 g  or  ≥22 wksTotal  births≥500 g  or  ≥22 wks×1000,FM=Fetal  deaths≥500 g  or  ≥22 wksTotal  births≥500 g  or  ≥22 wks×1000,ENM=Early  neonatal  deaths  or  ≥500 g  or  ≥22 wksLB≥500 g  or  ≥22 wks×1000.

WHO recommended definition for international comparison:

(2) PNM=Fetal  deaths  and  early  neonatal  deaths≥1000 g  or  ≥28 wksTotal  births≥1000 g  or  ≥28 wks×1000,FM=Fetal  deaths≥1000 g  or  ≥28 wksTotal  births≥1000 g  or  ≥28 wks×1000,ENM=Early  neonatal  deaths  or  ≥1000 g  or  ≥28 wksLB≥1000 g  or  ≥28 wks×1000.

Following the year 2001, according to the new methodology, the calculations for PNM and FM based on reports from the maternities have been obtained according to the national and international reporting criteria [13]. However, for the purpose of obtaining the vital statistics, all dead LB, independently of BW or GA, have been included in infant mortality.

Maternal mortality data from medical death records in CIPH were matched with CBS vital statistics data. The record of each death during pregnancy, childbirth, and puerperium was verified together by experts from CIPH and CSPM in order to get complete and reliable data. The WHO defines maternal death as the death of a woman while pregnant or within 42 days of termination of pregnancy, irrespective of the duration and site of the pregnancy, of any cause related to or aggravated by the pregnancy or its management but not of accidental or incidental causes. This definition allows identification of maternal deaths based on their causes as either direct or indirect. The direct obstetric deaths are those resulting from obstetric complications of the pregnant state (pregnancy, delivery, and postpartum period), from interventions, omissions, or incorrect treatment, or from a chain of events resulting from any of the above. The indirect obstetric deaths are those resulting from previous existing disease or diseases that develop during pregnancy and which were not due to direct obstetric causes but were aggravated by physiological effects of pregnancy [14]. The WHO Conference agreed that since the number of LB was more universally available than the number of total births, it should be used as the denominator in the ratios related to maternal mortality [2, 14]. Therefore, the maternal mortality ratio (MMR) in Croatia has been calculated including direct and indirect causes of woman death in pregnancy, delivery, or puerperium on 100,000 LB [13].

Data for process indicators (antenatal visits, US, and CS) have been obtained from maternities and CS proportions on 100 LB compared with WHO Health For All (WHO-HFA) database indicators.

## 3. Results

### 3.1. Perinatal Outcome Indicators

#### 3.1.1. Perinatal, Fetal, and Early Neonatal Mortality by Birth Weight Subgroups (≥500 g and ≥1000 g)

In the period 2001–2014, PNM for all ≥500 g BW total births was reduced by 30.6%, from 9.8‰ to 6.8‰, FM for all ≥500 g by 32.1%, from 5.6‰ to 3.8‰, and ENM by 28.6%, from 4.2‰ to 3.0‰. In the same period of time, PNM for all ≥1000 g BW total births was reduced by 42.5% (from 7.3‰ in 2001 to 4.2‰ in 2014). The FM was reduced by 35.6% (from 4.5‰ to 2.9‰) and ENM by more than half (53.6%, from 2.8‰ to 1.3‰), with slight variations in the rates over the years (Table 2).

#### 3.1.2. Perinatal, Fetal, and Early Neonatal Mortality by Gestational Age Subgroups (≥22 wks and ≥28 wks)

In the period 2001–2014, PNM in all ≥22 wks GA group was reduced by about one-quarter (27.6%, from 9.8‰ in 2001 to 7.1‰ in 2014). FM was reduced by 29.8% (from 5.7‰ to 4.0‰) and ENM by 26.2% (from 4.2‰ to 3.1‰). In the same period of time, PNM in all ≥28 wks GA was reduced by 40.6% (from 7.4‰ to 4.4‰), FM in all ≥28 wks GA by 36.1% (from 4.7‰ to 3.0‰), and ENM by 44.6% (from 2.7‰ to 1.5‰) (Table 3).

**Figure 1:**
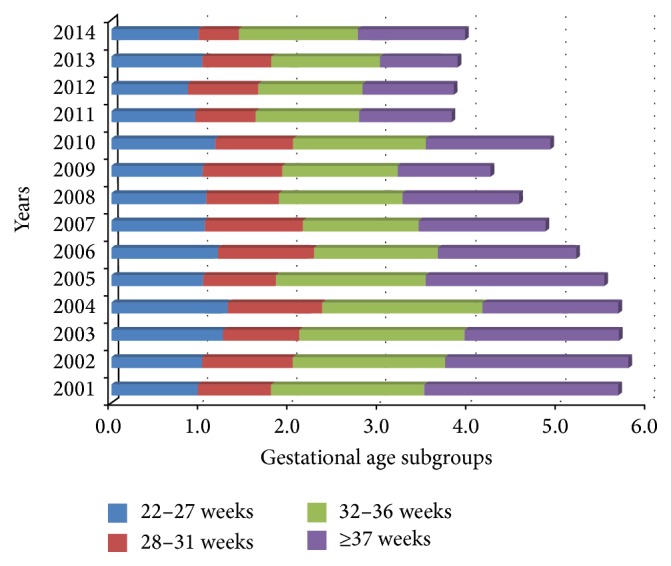
Fetal mortality attributed to gestational age subgroup (per 1,000 total births).

Figure 1 reports FM attributable to each of the four GA groups: 22–27; 28–31; 32–36; and ≥37 wks. This figure illustrates the impact of FM subgroup differences on overall rates. FM ranged from 1.2‰ to 1.8‰ in 32–36 wks GA subgroup and from 0.9‰ to 2.2‰ in 37–41 wks GA subgroup, for both subgroups more than a half of overall FM throughout the period 2001–2014.

**Figure 2:**
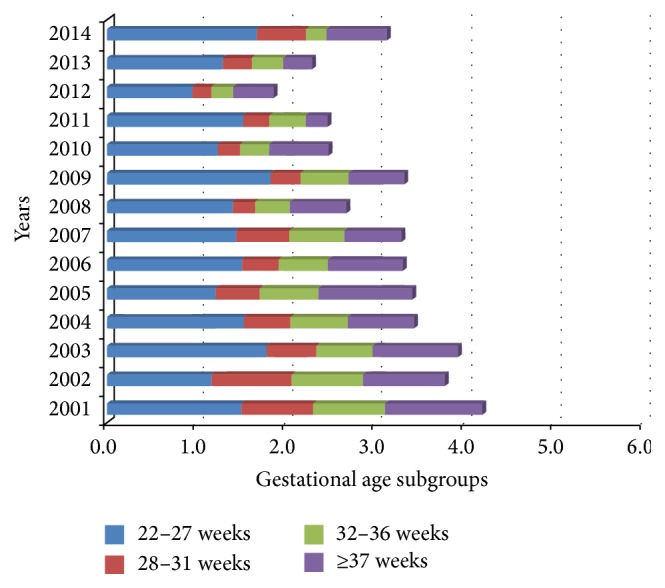
Early neonatal mortality attributed to gestational age subgroup (per 1,000 live births).

ENM was the highest in 22–27 wks GA subgroup, representing more than one-third to more than a half of the total of all early neonatal deaths in the period 2001–2014. ENM ranged from 1.0‰ to 1.8‰ in this GA (Figure 2).

### 3.2. Maternal Mortality

Table 4 reports MMR related to the direct and indirect obstetric causes for the period 2001–2014. Total MMR varied from 2.4 to 14.3/100,000 LB. In the period 2010–2014, the decreasing trend in direct obstetrical causes could be observed. In the period 2001–2014, the direct obstetric deaths due to pregnancy, labor, or puerperium caused 63.8% of all maternal deaths. The indirect obstetric deaths due to maternal chronic diseases, malignant diseases, and other causes unrelated to pregnancy, labor, or puerperium caused the remaining 36.2% of all maternal deaths.

### 3.3. Perinatal Process Indicators

#### 3.3.1. Antenatal Visits and Ultrasound Examinations

**Figure 3:**
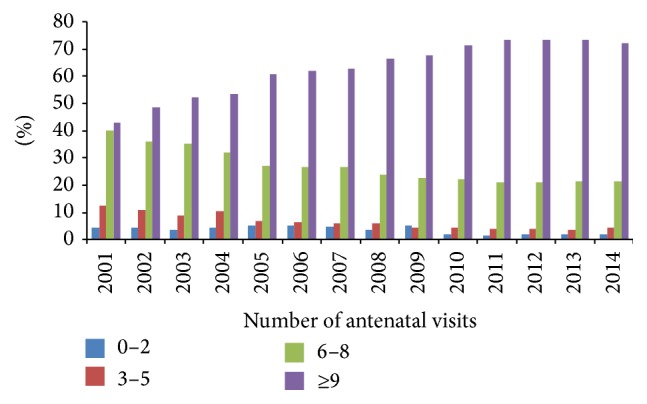
Distribution of pregnant women in relation to antenatal visits in the period 2001–2014.

Figure 3 reports the proportion of pregnant women with 0–2, 3–5, 6–8, and ≥9 antenatal visits. The percentage of pregnant women with ≥9 visits increased from 43.0% in 2001 to 72.3% in 2014, followed by other subgroups' proportion decrease.

**Figure 4:**
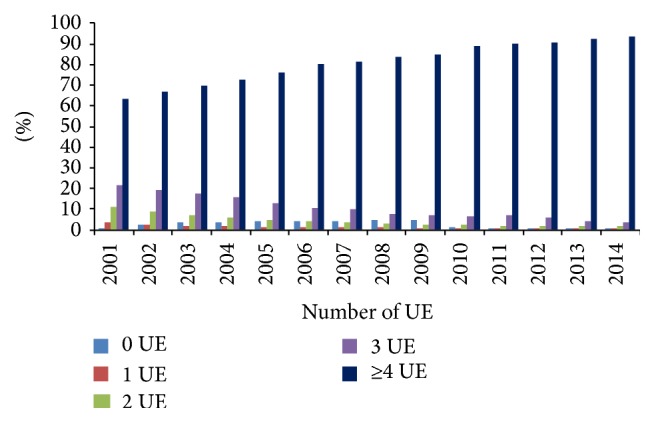
Distribution of pregnant women by ultrasound examination frequency in the period 2001–2014.

Figure 4 reports the proportion of pregnant women with 0, 1, 2, 3, and ≥4 US in pregnancy. The percentage of pregnant women with ≥4 visits increased from 63.1% in 2001 to 93.4% in 2014, followed by other subgroups' proportion decrease.

#### 3.3.2. Caesarean Section

**Figure 5:**
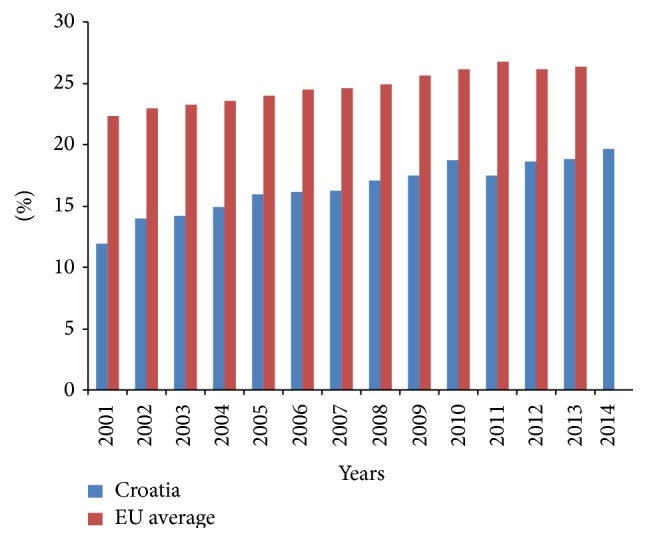
The proportion of caesarean sections in Croatia and EU average per 100 live births in the period 2001–2014.

The frequency of CS is continuously rising.  Figure 5 reports the comparison with EU average.

## 4. Discussion

The key indicators for perinatal health care quality assessment in Croatia were analyzed after introducing new reporting criteria in routine health statistics on national level for monitoring PNM, FM, and ENM in 2001. The changes in reporting criteria have enabled us to have a deeper insight into PNM, FM, and ENM trends in births <1000 g BW and <28 completed wks GA. According to reporting criteria for international comparisons and WHO-HFA indicators, PNM and FM in Croatia were below EU average [15]. However, this study showed that the inclusion of perinatal deaths <1000 g BW and <28 completed wks GA considerably changes picture about perinatal outcomes in Croatia. Furthermore, the study showed that the highest ENM was in 22–27 wks GA, by more than third to more than a half of the total ENM in the period 2001–2014, which is reflected in the increase in total PNM, in particular in 2013-2014.

Another important finding of this study was that, according to antenatal visits and US examinations which are prenatal care measures, the highest number of mothers was included in the optimal number of examinations in accordance with national recommendations [16]. This implies that some other factors like perinatal health care organization could have influenced ENM and PNM trends in 22–27 wks GA in Croatia and should be additionally investigated.

The mortality indicators are considered the most important measures of perinatal outcome, encouraging the health care professional efforts to prevent avoidable deaths. The data reliability depends on credible reporting and births and deaths recording process. The numerous researches have been emphasizing the problem of stillbirths and early neonatal deaths underreporting and misclassification [17–27]. The civil registration systems from many countries provide only basic information related to numbers of births and deaths and registration is required by law. However, the majority of civil registers do not collect birth or perinatal death data according to BW or GA. As in majority of countries, the main source of perinatal mortality indicators in Croatia was civil registration system, which was based on the birth and death certificates from CBS. According to the CBS methodology, FM is calculated as a number of stillbirths after 28th completed wks of pregnancy on 1,000 total births, irrespective of GA, up to the year 2001. ENM is calculated as a number of newborns who died in the first 168 hours (7 days) of life on 1,000 LB, irrespective of BW or GA. Whereas CBS does not collect data according to WHO and PERISTAT recommendations, it was impossible to carry out precise perinatal monitoring and international comparisons. Therefore, CSPM and CIPH introduced perinatal monitoring according to reports from maternities and developed new medical birth and perinatal death certificates by BW and GA, implementing their usage in the national routine health statistics system which covers more than 99% of births and perinatal deaths. However, the new monitoring system based on individual records needed a few years for developing and improving [13, 17]. Since 2001, Croatia has been providing the data about all perinatal deaths ≥1000 g to WHO-HFA for PNM calculations according to criteria for international comparison. For the purpose of the national analyses and evaluation of perinatal health care, both rates (according to BW ≥ 500 g and ≥ 1000 g) are used.

A similar reporting problem was detected in many European countries, preventing the perinatal, neonatal, postneonatal, and infant mortality comparisons by the BW or GA subgroups per country [22]. The health statistics systems differ in data collection methodology and area of coverage. Many countries use some form of linkage procedure to merge data from different sources. In international databases such as WHO-HFA and the Statistical Office of the European Communities (Eurostat) database, these indicators can be found to be related to the different methodologies: for WHO-HFA database countries, the perinatal mortality indicator for BW group ≥1000 g or group ≥28 wks of GA is provided, while for Eurostat, it is according to different national statistical offices data for vital statistics registration. These data are not sufficient for perinatal outcome measures as opposed to the PERISTAT subgroup and GA division due to the fact that more than 70% of perinatal deaths in developed countries are connected with preterm birth and low BW [17–19, 21, 22, 28]. The lack of BW and GA data for late neonatal and postneonatal deaths hinders the analyses of the long-term consequences caused by ELBW and/or GA: physical, neurological, and cognitive impairments.

PNM, FM, and ENM by BW and GA have been regularly analyzed and discussed at annual national perinatal mortality conferences as a form of perinatal surveillance with the basic aim of preventing unfavorable perinatal outcomes [10]. Croatian PNM, calculated by WHO-HFA methodology criteria of BW ≥ 1000 g, seems to be lower than European Union (EU) average, amounting to below 5‰ for the period of 2007 onwards [15]. FM rates were 5.7‰–4.0‰ for ≥22 wks in the period 2001–2014, mildly decreasing from 2001 to 2014. In comparison with the PERISTAT survey data for 2010, the European countries range from 2.6‰ to 8.9‰ [29]. The first Croatian data originated in the year 1950, indicating FM rate of 17.8‰, which gradually decreased to today's value [10]. ENM rates were 4.4–3.1‰ for ≥22 wks in the period 2001–2014, decreasing in 2012 and increasing in the years 2013 and 2014. In comparison with the PERISTAT survey data for 2010, the European countries range from 1.0‰ to 4.0‰, but mostly in the scope from 1.5‰ to 2.0‰ [29]. The first officially published ENM rate in Croatia was 27.7‰ in the year 1950 with substantial decrease, especially after 1996 when FM rate was surpassed [10]. The perinatal health in Croatia, measured by PNM, FM, and ENM, has improved considerably in recent decades with the evident increase in ENM during the last two years, especially in 22–27 GA subgroup, which represents the cause for concern and requires detailed new analyses. According to other national studies, the increased number of ELBW newborns, mostly from multiple pregnancies, led to the rise of ENM resulting with the consequent rise of PNM over the last two years [12, 30–32].

The increase in the number of <1500 g BW newborns, LB, and deaths directs the perinatal health care endeavor towards the prevention, early diagnosis, and appropriate treatment of threatened early preterm labors, harmless delivery of those children, and thereafter appropriate treatment of those newborns in the NICUs [10, 23]. This may be achieved by implementing a regional organization of perinatal health care according to evidence-based studies and observations [33–43]. The routine perinatal health monitoring system is an important tool which enables the health care planning process in accordance with the requirements for appropriate level of health care, including human resources and adequate equipment. In order to improve the structure of Croatian perinatal health care system, all maternities and neonatal units are organized in a network, regionalized according to the professional guidelines [44]. However, the network is not officially confirmed by the Ministry of Health of the Republic of Croatia. The pregnant women are referred, as well as postpartum sick newborns, to the facilities of appropriate level, according to maternal/infant health condition. The referrals are mainly towards the maternities with NICUs, level III, situated in own perinatal region. The most complicated pregnancies and newborns can be referred to the National Center of Perinatal Medicine or to the National Center of Neonatal Intensive Medicine (level IV). The transfer of the sick newborns is organized as “one-way transport” [44].

The pregnancy and childbirth still involve risk for pregnant women and their babies and health in the perinatal period, while remaining an important public health priority. Although poor outcomes are increasingly rare, mothers in Europe still die in childbirth (5–15 women per 100,000 LB) [45]. Not only does MMR represent a key perinatal health outcome, but also it indicates the quality of obstetrical care, since many direct maternal deaths are associated with substandard care. The analysis of maternal deaths revealed that one-third of them are avoidable. The indicators of maternal mortality are extremely sensitive to underreporting, both in developing and in developed countries [46, 47]. The ascertainment of maternal deaths requires an effort by governments to ensure that deaths during or within one year after pregnancy are identified on death certificates or using other measures. Their precise registration depends on cause of death coding rules.

MMR for Croatia displays the substantial variation over time and the average is 8.1/100,000 LB during the period of 14 years, slightly higher than EU average considering the last few years. In 1954 (first known data), maternal mortality was 168/100,000 LB, rapidly decreased in the period 1960–1980, afterwards showing the values below 10/100,000 LB [48]. Following the introduction of the new reporting criteria and registration in the year 2001, the death causes of women in pregnancy, labor, and 42 days after labor are double-checked by CIPH and CSPM. The increase of the overall maternal deaths might have been caused by the fact that CIPH and CSPM have been including the indirect causes of maternal death as a part of the overall maternal deaths count since 2001. Up to the year 2001, Croatia reported only direct obstetrical maternal deaths.

The number of clinical visits and of US examinations of pregnant women is continuously increasing. The recommendations of CSPM, officially adopted and completely financed by Croatian Institute of Health Insurance, are 10 clinical visits per healthy pregnant woman and 3 US examinations [44]. The low proportion of pregnant women without adequate antenatal care and ultrasound examinations represents the indicators of prenatal care availability quality.

In view of the ongoing debates about the safest path to delivery, there is not yet clear consensus achieved. The spontaneous deliveries represented the majority of births in all countries, but the proportion of CS has been increased in the majority of European countries [15]. The rise of CS in Croatia, in comparison to the majority of European and other countries, is relatively mild. In relation to USA and some European countries, there is a certain lag period present [49, 50]. There is an endeavor to stop further CS rise. The measurements of differences in mode of delivery under different circumstances including breech presentation, previous CS, parity, and multiple gestation pregnancies would offer better insight into necessity, risks, and benefits in specific circumstances.

The strength of this study is the perinatal mortality audit based on national routine health statistics which enables calculation of PNM, FM, and ENM rates adjusted for GA and BW for the whole population. The results can be considered as fairly reliable and representative for the entire country. Our review of perinatal mortality outcomes related to BW and GA specific mortality rates over the period of 14 years and comparisons with PERISTAT 2010 report can be considered a way to improve the health care process for all pregnant women and their newborns. It provides an opportunity to learn from adverse events, identifying and analyzing them and providing the future preventive measures.

The limitations of this study include the lack of the other perinatal health care indicators required for detailed insight into the provided perinatal health care, as well as perinatal morbidity outcomes with long-term physical, neurological, and cognitive impairment.

## 5. Conclusions

The perinatal health audit in Croatia has been improved after introducing recommended reporting criteria by WHO and PERISTAT which enable comparison in perinatal outcome with other countries. The outcome of this research provides an opportunity to identify problems and to prepare the plan for perinatal health care improvement. The perinatal monitoring system should be further improved while analyzing other perinatal indicators, except for the presented few core outcomes and process indicators, in order to achieve more complete image of perinatal care effectiveness and availability. The comprehensive perinatal health monitoring represents the basis for the perinatal quality assessment.

## Figures and Tables

**Table 1:** Total births, fetal, early neonatal and perinatal deaths in Croatian maternities in the period 2001–2014.

Year	Total births ≥22 wks numbers	Fetal deaths ≥22 wks numbers	Early neonatal deaths ≥22 wks numbers	Perinatal deaths ≥22 wks numbers
2001	41,487	235	173	408
2002	40,493	234	152	386
2003	40,013	227	156	383
2004	40,759	231	139	370
2005	43,030	237	146	383
2006	41,964	218	138	356
2007	42,456	195	139	334
2008	44,315	202	118	320
2009	45,071	191	149	340
2010	43,842	215	108	323
2011	41,556	158	102	260
2012	42,074	161	78	239
2013	40,319	156	92	248
2014	39,977	158	125	283

**Table 2:** Perinatal, fetal, and early neonatal mortality rates (‰) in total births' birth weight ≥500 g and birth weight ≥1000 g in Croatia in the period 2001–2014.

Year	PNM ≥ 500 g (‰)	PNM ≥ 1000 g (‰)	FM ≥ 500 g (‰)	FM ≥ 1000 g (‰)	ENM ≥ 500 g (‰)	ENM ≥ 1000 g (‰)
2001	9.8	7.3	5.6	4.5	4.2	2.8
2002	9.3	6.9	5.6	4.3	3.7	2.6
2003	9.5	6.3	5.7	4.1	3.8	2.2
2004	8.7	5.8	5.3	3.9	3.4	1.9
2005	8.8	6.4	5.4	4.2	3.4	2.2
2006	8.3	5.3	5.1	3.4	3.2	1.9
2007	7.8	4.9	4.5	3.1	3.2	1.8
2008	7.0	4.6	4.4	3.2	2.6	1.4
2009	7.2	4.4	4.2	3.0	3.0	1.5
2010	7.2	4.7	4.8	3.5	2.4	1.2
2011	5.9	3.5	3.6	2.5	2.3	1.0
2012	5.4	3.6	3.6	2.7	1.7	0.9
2013	5.8	3.5	3.6	2.5	2.2	1.0
2014	6.8	4.2	3.8	2.9	3.0	1.3

**Table 3:** Perinatal, fetal, and early neonatal mortality rates (‰) in gestational age subgroups ≥22 weeks and ≥28 weeks in Croatia in the period 2001–2014.

Year	PNM ≥ 22 wks (‰)	PNM ≥ 28 wks (‰)	FM ≥ 22 wks (‰)	FM ≥ 28 wks (‰)	ENM ≥ 22 wks (‰)	ENM ≥ 28 wks (‰)
2001	9.8	7.4	5.7	4.7	4.2	2.7
2002	9.5	7.4	5.8	4.8	3.8	2.6
2003	9.6	6.6	5.7	4.4	3.9	2.1
2004	9.1	6.3	5.7	4.4	3.4	1.9
2005	8.9	6.7	5.5	4.5	3.4	2.2
2006	8.5	5.8	5.2	4.0	3.3	1.8
2007	7.9	5.5	4.6	3.6	3.3	1.9
2008	7.2	4.8	4.6	3.5	2.7	1.3
2009	7.5	4.7	4.2	3.2	3.3	1.5
2010	7.4	5.0	4.9	3.8	2.5	1.2
2011	6.3	3.8	3.8	2.9	2.5	0.9
2012	5.7	3.9	3.8	3.0	1.9	0.9
2013	6.2	3.9	3.9	2.9	2.3	1.0
2014	7.1	4.4	4.0	3.0	3.1	1.5

**Table 4:** Maternal deaths and maternal mortality ratios related to the direct and indirect obstetric causes/100,000 live births.

Year	MD: all causes		MD: direct obstetric causes		MD: indirect obstetric causes	
	Numbers	MMR	Numbers	MMR	Numbers	MMR
2001	1	2.4	1	2.4	0	0
2002	4	10.0	4	10.0	0	0
2003	3	7.6	3	7.6	0	0
2004	3	7.4	2	5.0	1	2.5
2005	3	7.1	1	2.4	2	4.7
2006	4	9.7	2	4.8	2	4.8
2007	6	14.3	3	7.2	3	7.2
2008	3	6.9	1	2.3	2	4.6
2009	6	13.5	6	13.5	0	0
2010	4	9.2	1	2.3	3	6.9
2011	4	9.7	3	7.3	1	2.4
2012	3	7.2	1	2.4	2	4.8
2013	2	5.0	1	2.5	1	2.5
2014	1	2.5	1	2.5	0	0

2001–2014	47	8.1	30	5.2	17	2.9
